# Editorial for the Special Issue on the Insights and Advancements in Microfluidics

**DOI:** 10.3390/mi8080254

**Published:** 2017-08-17

**Authors:** Say Hwa Tan, Heng-Dong Xi, Weihua Li

**Affiliations:** 1 Queensland Micro- and Nanotechnology Centre, Griffith University, 170 Kessels Road, Brisbane, QLD 4111, Australia; sayhwa.tan@griffith.edu.au; 2 School of Aeronautics, Northwestern Polytechnical University, 127 West Youyi Rd., Xi’an 710072, China; hengdongxi@nwpu.edu.cn; 3 School of Mechanical, Materials and Mechatronic Engineering, University of Wollongong, Wollongong, NSW 2522, Australia

We present a total of 19 articles in this special issue of *Micromachines* entitled, ”Insights and Advancements in Microfluidics.” Among the 19 articles, two perspectives, eight reviews, and nine research articles were solicited from leading researchers, pioneers, and emerging investigators. The topics covered in this issue ranges from biology, chemistry, and physics to the intersection of engineering, optics, and material sciences. As editors for this issue, we are both gratified and extremely thankful for the overwhelming responses and contributions from our fellow colleagues within the field of microfluidics.

The special issue is themed to provide both insights and advancements in microfluidics. This well-timed issue touches on a field which has evolved tremendously in the last few decades. Professor Yanyi Huang from Peking University of China provided his unique insight and perspective on digital polymerase chain reaction (PCR) [1]. In his article, an informative guide was provided on the proper designing rules of digital PCR at the micro-scale. Professor Guoqing Hu from the Chinese Academy of Sciences, Beijing, China, provided his astute insights and perspective on particle manipulation based on hydrodynamic effect [2]. His article summarizes both the progress and fundamental mechanisms in particle manipulation using elasto-inertial microfluidics.

In the eight reviews articles, different branches and sub-branches of microfluidics were presented and comprehensively reviewed. These include microfluidic sensing, liquid handling, optofluidics, the use of microfluidics in cytotoxicity, Janus micro-motors, single-cell impedance cytometry, droplets, and polymer microfluidics. We were extremely fortunate to receive contributions from both Professor Dongqing Li and Professor Nam-Trung Nguyen. Both are leading pioneers and extraordinary visionary leaders in the field of microfluidics. Professor Li et al. [3] reviewed the basic theories in both microfluidic and nanofluidic resistive pulse sensing (RPS). His article focuses on the latest developments in the last six years. Future research direction and challenges in this area are also outlined in the review. Professor Nguyen et al. [4] discussed the recent advances and future perspective on microfluidic liquid handling. The first part of the review covers two main and opposing applications of liquid handling in continuous-flow microfluidics: mixing and separation. The second part focuses on various digital microfluidic strategies based on both droplets and liquid marbles. The applications of the emerging field of liquid-marble-based digital microfluidics are also highlighted in the article. Song et al. [5] provided an overview on the recent development of optofluidics. They discussed the critical challenges that hamper the transformation of optofluidic technologies from lab-based procedures to practical usages and commercialization. Priest et al. [6] reviewed the different microfluidic chips that can used for toxicity screening. Li et al. [7] discussed the self-propulsion of a platinum–silica (Pt–SiO_2_) spherical Janus micro-motor (JM). Their paper reviews two distinct mechanisms, self-diffusiophoresis and microbubble propulsion, and demonstrates that the physical principles of these mechanisms can be used to fulfill many novel functions. Petchakup et al. [8] discussed the topic of microfluidic-based single-cell impedance cytometry. Their article reviews the recent developments, applications, and discusses the future direction and challenges in this field. Wang et al. [9] re-visited the use of droplet-based microfluidics for the production of both micro and nano particles. Tsao et al. [10] discussed simple, low-cost methods to fabricate polymer-based microfluidics devices. Their paper provides an overview of the different micro-fabrication methods and discusses the current challenges of this research.

The nine research articles provides advancement techniques which can be categorized into two distinct classes: device innovation and fluid dynamics. In device innovation, Shui et al. [11] used the vacuum airbag laminator (VAL) to fabricate large-area and high-throughput PDMS microfluidic devices. The proposed fabrication method can achieve a high bond strength with a maximum breakup pressure of about 739 kPa. Lim and Kim et al. [12] proposed a new method to fabricate an all-glass bifurcation microfluidic chip using an amorphous carbon (AC) mold. The device is then used for blood plasma separation. Koh et al. [13] used a modified xurography method to fabricate a low-cost microfluidic device which can be used for both droplet fission and encapsulation. Lim et al. [14] devised a new bonding method which can be used to enhance the fabrication of thermoplastic microfluidic devices. This method combines an interference fit with a thermal treatment at a low pressure. Sui et al. [15] demonstrated a new microfluidic chip that can be used for rapid capture and analysis of airborne staphylococcus. The whole analysis takes less than 5 h and has a detection limit down to about 27 cells. Choi et al. [16] presented a simple but yet effective approach for facile, on-demand reconfiguration of microfluidic channels using flexible polymer tubing. Both microparticle separation and fluid mixing were successfully implemented by reconfiguring the shape of the tubing. 

In fluid dynamics, Tsao et al. [17] used a cyclic olefin copolymer (COC)-based microfluidic device to investigate the flow behavior in a fractured porous medium. His results show that the flow resistance in the main channel with a large radius was higher than that in the surrounding area with small pore channels when the injection or extraction rates were low. When the flow rates were increased, the extraction efficiency of the water and oil in the mainstream channel did not increase monotonically because of the complex two-phase-flow dynamics. Zhao et al. [18] used numerical simulations to investigate the dynamic characteristics of electro-osmosis. His investigation using the finite element method shows that the electro-osmotic flow of power-law fluids under an AC/DC combined driving field is enhanced when compared to a pure DC electric field. Qin et al. [19] used a Y-shaped microfluidic device to study the combined effect of wall shear stress (WSS) and adenosine triphosphate (ATP) signals on intracellular calcium dynamics in vascular endothelial cells (VEC). Both numerical simulation and experimental studies verified the approach. The experimental results also suggest that a combination of WSS and ATP signals (rather than a WSS signal alone) play a more significant role in VEC Ca^2+^ signal transduction induced by blood flow.

Last but not least, we wish to thank all authors who submitted their papers to this special issue. We would also like to acknowledge all the reviewers whose careful and timely reviews ensured the quality of this special issue.
